# Supplementary material for: A 14,000-year-old genome sheds light on the evolution and extinction of a Pleistocene vulture
Source: Commun Biol. 2022 Aug 23;5:857. doi: 10.1038/s42003-022-03811-0 (PMC9399080; doi:10.1038/s42003-022-03811-0)
Supplement: Supplementary file 2 — Supplementary Information [file 42003_2022_3811_MOESM2_ESM.pdf]

Supplementary information

A 14,000-year-old genome sheds light on the evolution and extinction of a Pleistocene vulture

Per G.P. Ericson [1*], Martin Irestedt [1], Dario Zuccon [2], Petter Larsson [1,3], Jean-Luc Tison [4], Steven D. Emslie [5], Anders Götherström [3,6], Julian P. Hume [7], Lars Werdelin [8], Yanhua Qu [1,9*]

[1] Department of Bioinformatics and Genetics, Swedish Museum of Natural History; P.O. Box 50007, SE-10405, Stockholm, Sweden

[2] Institut de Systématique, Evolution, Biodiversité (ISYEB), UMR7205 CNRS MNHN UPMC EPHE Sorbonne Université, Muséum National d'Histoire Naturelle; 75005 Paris, France

[3] Centre for Palaeogenetics; Stockholm, Sweden

[4] Department of Laboratory Medicine, Örebro University Hospital; Södra Grev Rosengatan, SE-70185 Örebro, Sweden

[5] Department of Biology and Marine Biology, University of North Carolina; Wilmington, 601 S. College Road, Wilmington, NC 28403, USA

[6] Department of Archaeology and Classical Studies, Stockholm University; SE-10691 Stockholm, Sweden

[7] Bird Group, Department of Life Sciences, Natural History Museum; Akeman St., Tring, Herts, UK

[8] Department of Palaeobiology, Swedish Museum of Natural History; P.O. Box 50007, SE-10405, Stockholm, Sweden

[9] Key Laboratory of Zoological Systematics and Evolution, Institute of Zoology, Chinese Academy of Sciences; Beijing, 100101, China

* per.ericson@nrm.se; quyh@ioz.ac.cn

Contents:

Supplementary Notes 1 to 7

Supplementary Methods 1 to 2

Supplementary Tables 1 to 6

Supplementary Figures 1 to 5

Supplementary References

Supplementary Note 1. Paleontological locality

In 1904-05, the Swedish ethnographer Erland Nordenskiöld collected numerous mammal and bird fossils from the cave Casa del Diablo, situated at >3800 m a.s.l. in the southern Peruvian Altiplano (1). Fossils from the cave have previously been dated to between 23 and 12.8 cal kyr BP (calibrated thousands of years before present, table S1) and the megafauna includes several extinct groups such as ground sloths (*Megatherium*), sloths (*Diabolotherium*) and horses (*Hippidion*) along with extant species such as the camelids *Lama guanicoe* and *Vicugna vicugna* (2, Supplementary Figure 1). The material is stored at the Department of Paleobiology, Swedish Museum of Natural History in Stockholm, Sweden. The vulture specimens studied herein were identified during an ongoing study of the paleontological bird material from the Casa del Diablo cave (Per Ericson in prep.). The identification of the vulture material was done using collections of fossil and recent vultures at the National Museum of Natural History (Smithsonian Institution) in Washington, the Natural History Museum of Los Angeles County, and the La Brea Tar Pits Museum (George C. Page Museum) in Los Angeles.

Supplementary Note 2. Systematic paleontology

Class AVES

Order ACCIPITRIFORMES

Family CATHARTIDAE

[*Coragyps*] *occidentalis* (L. Miller, 1909)

Material. Four specimens (NRM PAL-PZ A312-315) probably deriving from a single individual: complete left coracoid, distal right ulna, almost complete right carpometacarpus, and proximal right femur (Fig. 1b).

Measurements (mm). Coracoid (A312); length, head to sternal facet 65.6, breadth across triosseal canal 12.1. *Ulna* (A313); greatest length c. 175, transverse breadth through middle of shaft 8.4. *Carpometacarpus* (A314); length to distal end of metacarpal 83.8, breadth of proximal trochlea 9.6, least breadth of shaft of metacarpal II 7.6. *Femur* (A315); least transverse breadth of shaft 10.7.

Description. The Casa del Diablo specimens agree with *Coragyps* and differ from *Cathartes* in numerous characters, among which the most clear-cut are reported here. In the coracoid, cotyla scapularis is well excavated and not almost flat as in *Cathartes* (it may even be convex in some individuals of *Cathartes*). Processus procoracoideum is positioned at about two-thirds of the total length between angulus medialis and the tip of processus acrocoracoideum. In *Cathartes*, this process is positioned almost at the middle of this length. In medial view, processus acrocoracoideum differs between *Coragyps* and *Cathartes* in being more pronounced in the latter genus. In this character, the Casa del Diablo specimen agrees with *Coragyps*. In the proximal ulna, the distance between the tip of the olecranon and the distal border of the tuberculum bicipitale is short in relation to the overall size of the bone, as in *Coragyps*. In the distal end of the ulna, the border of the condylus dorsalis forms a smooth curve as in *Coragyps* and is not almost rectangular as in *Cathartes*. In the proximal carpometacarpus, fossa infratrochlearis is deeply excavated as in *Coragyps*. In the proximal femur, we have found no consistent character to separate the genera, although crista trochanteris is more rounded in lateral view in most specimens of *Cathartes*, but always somewhat triangular in *Coragyps*. In this character, the fossil specimen again agrees with *Coragyps* and differs from *Cathartes*.

Supplementary Note 3. Do the new Peruvian fossils belong to Coragyps*?*

Morphological data – Comparisons of this fossil have been made with all extant species of New World vultures using the collections of the natural history museums in Stockholm, Copenhagen, Los Angeles and Washington. Early on it was clear that the fossils derive from a member of the genus *Coragyps*, of which *C. atratus* is the only extant member. As described in the Systematic paleontology section (Supplementary Note 2), the specimens agree with *Coragyps* and differ from the closely related *Cathartes* in numerous characters of which the clearest are:

- Coracoid: Cotyla scapularis is well excavated and not almost flat as in *Cathartes* (in which it may even be convex in some individuals), the processus procoracoideum is positioned at about 66% of the total length between angulus medialis and the tip of processus acrocoracoideum. In *Cathartes*, this processus is positioned almost midway between these points. The processus acrocoracoideum, as seen in medial view, differs in *Coragyps* and *Cathartes* in that it is relatively much larger in the latter genus. In this character also, the Casa del Diablo specimen agrees with *Coragyps*.
- Ulna: The distance between the tip of the olecranon and the distal border of the tuberculum bicipitale is short in relation to the overall size of the bone, as in *Coragyps*. In the distal end, the border of the condylus dorsalis forms a smooth curve as in *Coragyps* and is not almost rectangular as in *Cathartes*.
- Carpometacarpus: Fossa infratrochlearis is deeply excavated as in *Coragyps*.
- Femur: no consistent characters to separate the genera were found, although the crista trochanteris is more rounded in lateral view in most specimens of *Cathartes* but always somewhat triangular in Coragyps. In this character the fossil specimen agrees with *Coragyps* and differs from *Cathartes*.

Molecular data – We conducted a phylogenomic analysis including genomes of ten species sampled from the clade Accipitrimorphae (*sensu* 3) to which the families Accipitridae, Pandionidae, Sagittaridae and Cathartidae belong, and three outgroups from the families Phasianidae, Ptilonorhynchidae, and Corvidae. Genomes of the following taxa were downloaded from GenBank (accession numbers in

parentheses): *Gallus gallus* (GCA000002315), *Cathartes aura* (GCA000699945), *Gyps himalayensis* (GWHBAOP00000000), *Gypaetus barbatus* (GWHBAOQ00000000), *Buteo japonicus* (GCA010312235), *Haliaeetus albicilla* (GCA000691405), *Sagittarus sepentarius* (GCA013399415), *Pandion haliaetus* (GCA013401275), *Gymnogyps californianus* (GCA018139145), *Amblyornis subalaris* (GCA018881555), and *Corvus cornix* (GCA000738735). In addition, we included the *de novo* genome of *Coragyps atratus* from the present analysis and the genome of [*Coragyps*] *occidentalis* sequenced here. We used BirdScanner (see Material and Methods) to extract and align 892 exons (the alignments sum up to 567 kb). We used BEAST2 with a general time-reversible (GTR) model for nucleotide substitutions with 5 gamma rate categories (empirical base frequencies and free rates), a relaxed log-normal clock, and a birth–death tree prior. The MCMC chain was set to 50 million generations sampling every 1,000 generations and we used Tracer to assure that an adequate (~200) effective sample sizes (ESS) had been reached for all parameters indicating proper MCMC mixing and convergence. The molecular analysis recovered phylogenetic relationships in broad agreement with previous analyses (3-6) and confirmed the sister-group relation between the Casa del Diablo fossils and *Coragyps atratus* (Fig. 2a).

Supplementary Note 4. Is occidentalis *only an upscaled version of* atratus*?*

It has long been acknowledged that *occidentalis* is not just an isometric upscaling of *atratus*; they also differ in their body proportions. Fisher (7) studied the skulls of *occidentalis* (81 crania, 18 rostra and 4 incomplete mandibles) and *atratus* (n=10). He found them to differ considerably in both size and proportions:

"The Black Vulture of the Pleistocene certainly represents a species distinct from *C. atratus* of Recent times. In table 1 it may be observed that in all measurements of the skull *occidentalis* is larger. In length of premaxillary, length of premaxillary anterior to the nares, length of nares, and width of bill the ranges of the measurements do not overlap. These same characters are emphasized by the ratios in table 2.

Thus, the skull of *C. occidentalis* is significantly larger than that of *atratus* and the premaxillaries and nares are relatively longer. The ratios of temporal and postorbital width and cranial height to cranial length indicate that the Pleistocene vulture had a wider and somewhat higher brain case. The width at the frontonasal hinge is also greater, and bill depth is relatively less in *occidentalis*." (p.288)

"Aside from the quantitative characters there are important qualitative characters distinguishing *atratus* from *occidentalis*. In *occidentalis* the brain case is more inflated immediately anterior to the supraoccipital area; it is similar to *Cathartes aura* in this respect. The supraorbital edges are more excavated posteriorly and do not always form the characteristic straight line found in *atratus*. In some, the excavation approaches the depth found in *C. aura*. Because of the greater hinge width and interlachrymal width the crests are more nearly parallel in *occidentalis*.

The foramen magnum is larger and somewhat compressed vertically. The occipital processes are heavier, broader and smoother; this is reminiscent of those on *Breagyps*, but on a smaller scale. In *occidentalis* the pit on the postorbital process for muscle attachment is deeper and larger, and the posterodorsal corner of the masseter scar extends farther medially. The entire scar is deeper.

The proximal anterolateral surface of the lachrymal has only a very small opening compared to a large foramen in *C. atratus*. The nasal bridge is relatively heavier and the tip of the upper mandible is not hooked as much in *C. occidentalis*." (p.288)

Howard (8) also stressed the different proportions of *atratus* and *occidentalis*, this time in their postcranial skeleton. Based on more than 400 limb bones and coracoids of *occidentalis* from the Pleistocene of Rancho La Brea, Howard described *occidentalis* as having "relatively shorter, stouter tarsometatarsi and tibiotarsi, longer wings, and over-all larger body size" than *atratus* (p.127).

The observations by Fisher (7) and Howard (8) can be illustrated graphically with two ratio diagrams (Fig. 1c). The ratio diagram was introduced in paleontology by Simpson (9) to provide rapid and easy comparisons, of both size and body proportions, of different species. The logic behind this is that different ecological adaptations are likely to result in different body proportions and with a ratio diagram these can be directly compared. Our diagrams are modified from Simpson (9) as we want to keep the size difference between *atratus* and *occidentalis* by expressing each measurement in relation to the greatest length of humerus in *atratus* (a proxy for the overall size of this taxon). Through this we can compare both size and shape differences in the two taxa. With equal proportions between body parts, the two species would form parallel curves in the diagram, and these parallel curves would be shifted sideways according to differences in the size of these forms. If *occidentalis* was just an upscaled version of *atratus* the two lines in the ratio diagrams would be parallel, only shifted sideways depending on the difference in their average size. Instead, while *occidentalis* in some measurements is almost 120% larger than *atratus*, they are equal in size in other measurements. The different proportions observed in these two taxa suggest they were adapted to different lifestyles.

Supplementary Note 5. Could occidentalis *be the Pleistocene ancestor of* atratus *– a chronospecies?*

In the literature it is often suggested that *occidentalis* is the direct ancestor of *atratus* (7, 8, 10). However, the fossil record contradicts this:

a) It is evident that the large-sized *occidentalis* and the small-sized *atratus* were partly contemporary. For example, in the U.S. *occidentalis* is often referred to as the “Western Black Vulture” since it is well represented in Pleistocene deposits in, e.g., California, New Mexico and Nevada, while *atratus* specimens are predominantly recovered from Pleistocene deposits in the eastern regions of the U.S. where it is common today (Florida, Georgia and Maryland). In addition, the two finds in Peru show that these two taxa were contemporary also in South America: *atratus* has been recovered at the Pleistocene coastal site of Talara Tar Seeps in Peru (11), which is contemporaneous with the likewise Peruvian cave Casa del Diablo in the Andes where the *occidentalis* fossils studied herein were collected.

b) The fossil record (Figs. 1b and 1d*,* Supplementary Table 2) suggests that *atratus* is an older taxon than *occidentalis*. The oldest *atratus* fossils are dated between late Blancan and early Irvingtonian (1.9-1.6 my) (12), while other fossils have been dated to between 1.6 mya and 300 kya (12-14). Almost all (28 out of 29) finds of *occidentalis* are younger than 330 ky (Supplementary Table 2), the only exception is a distal humerus collected at the El Golfo site in Mexico. It is dated to between 1.35 and 0.781 my (15) and has been referred to as *occidentalis* (16) based on size (David W. Steadman pers. comm.). With a distal width to 25.6 mm (Steven D. Emslie pers. obs.) it does however fall within the range of *atratus* (8). Also, the old age of this specimen (at least 0.5 my older than the oldest certain *occidentalis*) raises questions about the reliability of using the measurements in (8) for taxonomic identification. Given the exceptionally old age of this specimen and its intermediate size between *occidentalis* and recent *atratus*, we question its assignment to *occidentalis* and regard it as *Coragyps* sp..

Supplementary Note 6. Is the large size of *occidentalis* an adaptation to high elevations?

One may first note that high elevations generally are colder than low elevations and it may therefore be logical to assume that such ecogeographical rules as Bergmann's and Allen's apply to mountainous populations. Bergmann's rule has been invoked to explain the large size of some Andean bird species, e.g., *Fulica gigantea* in relation to its sister species *F. cornuta* (Jon Fjeldså pers. comm.) and within the species-complex of *Cranioleuca* spinetails (31).

The taxon *atratus* occurs throughout the Americas and is almost exclusively found in lowland regions (below 283 m a.s.l., Fig. 2c). The geographic distribution of fossil *atratus* indicates that its distribution has been the same for many hundreds of thousands of years. Finds of large-sized *Coragyps* vultures are sometimes made at lowland sites, but no fossil of the small-sized *atratus* is known from elevations above 283 m (the average elevation for the 28 North American *occidentalis* finds is 1,083 m) (Fig. 1d). Precisely why a population of *atratus* would colonize extreme high elevation is unknown, but no fossil find suggests that this is something that happened repeatedly throughout its history. On the contrary, colonization and adaptation to a life at high elevations obviously represents a rare event.

Among New World vultures there are few opportunities for comparative studies. Several of the species are either distributed mainly in the lowlands (*Sarcoramphus papa, Cathartes burrovianus* and *Cathartes melambrotus*) or the highlands (*Vultur gryphus*), showing no or only minor geographic variation. Only one species, *Cathartes aura*, is distributed in both lowlands and highlands, allowing a study of how elevation may influence size in cathartid vultures. This species varies geographically in size in a way that is consistent with Bergmann's rule, i.e., individuals are generally larger at higher latitudes (32). In addition, there is also support for elevation being important for size because the largest subspecies in South America, *Cathartes aura jota*, is resident in the highlands from central and southern Colombia to southern Argentina (33). We believe that there is support for assuming that New World vultures increase in size with elevation. However, it remains to be shown what specific factors influence size in *Coragyps* vultures living at high elevations. Unfortunately, it is not possible to study this in the extant Black vulture *C. atratus*, as this taxon only occupies lower elevations. It should be

noted that, just as with *Cathartes aura*, there is tendency that individuals in the tropics of *Coragyps atratus* are smaller than those is temperate regions.

*Supplementary Note 7. Could the new Peruvian “*occidentalis*” be a similarly adapted, but different taxon than the North American “*occidentalis*”?*

Despite *occidentalis* being known from numerous fossils collected from at least 27 sites in North America, the find at Casa del Diablo is the first in South America. Given that *occidentalis* seemingly is a large-sized taxon that has evolved from a population of small-sized *atratus*, one may hypothesize that this could have taken place more than once. A genetic comparison with a North American specimen could answer this, but this may prove difficult. To our knowledge, no DNA has as yet been obtained from any fossil at an *occidentalis*-bearing locality, despite attempts targeting seemingly promising bones of large mammals.

Our assumption that the large Pleistocene *Coragyps* vultures in South and North America are the same taxon builds on the following evidence. The Casa del Diablo specimens have been compared directly with the specimens of *occidentalis* held by the Natural History Museum of Los Angeles County and the George C. Page Museum (Museum at La Brea Tar Pits). These museums hold numerous *occidentalis* specimens, including those collected at Rancho La Brea in California and San Josecito Cave in Mexico. The morphology of the Casa del Diablo specimens agrees completely with that of the North American *occidentalis* specimens with which they are compared. Specifically, the find from Casa del Diablo in Peru consists of four skeletal elements that provided five measurements that can be directly compared with the series for North American *occidentalis* specimens published by Howard (8). The measurements of the Casa del Diablo specimens all fall within the range of North American *occidentalis* (Supplementary Table 3). In addition, the body proportions of the Casa del Diablo specimens (marked with stars) are similar to those observed for the La Brea specimens (Supplementary Figure 3). Taken together, the most parsimonious explanation for the observation of two contemporary, large-sized *Coragyps* populations in North and South America is that they belong to the same taxon.

Supplementary Methods 1. Details about the aDNA laboratory procedures

We obtained ~50 mg bone powder of the femur (A215) using a Dremel high-speed multi-tool and DNA extraction was done according to the protocol in (17). We then built a double-stranded Illumina library according to Meyer & Kircher (18), with the additional step of treatment with USER enzyme to excise uracil residues resulting from post-mortem damage (19, 20). For blunt end repair, the DNA was incubated with 6U USER enzyme for 3 h at 37 °C, followed by the addition of 0.8 µl T4 DNA polymerase (Thermo Scientific), and incubation at 25 °C for 15 min and 12 °C for 5 min, and was then cleaned using MinElute (Qiagen, Hilden, Germany) spin columns and eluted in 20 µl EB Buffer following the manufacturer's protocol. Next, we performed an adapter ligation step where DNA fragments of the library were ligated to a combination of incomplete, partially double-stranded P5- and P7-adapters. This reaction was performed in a 40 µl reaction volume using 20 µl of blunted DNA from the clean-up step and 2 µl P5–P7 adapter mix (0.5 µM final concentration for each adapter) per sample with a final concentration of 1×T4 DNA ligase buffer, 5% PEG-4000, 5U T4 DNA ligase (Thermo Scientific). The sample was incubated for 30 min at room temperature and cleaned using MinElute spin columns as described above. Next, we performed an adapter fill-in reaction in 40 µl final volume using 20 µl adapter-ligated DNA with a final concentration of 1× Thermopol Reaction Buffer, 250 µM of each dNTP, 12U Bst Polymerase (Thermo Scientific), Long Fragments. The library was incubated at 37 °C for 20 min and heat-inactivated at 80 °C for 20 min.

This library was then amplified with double-indexed P5–P7 primers. The amplification was performed in 25 µl volumes with 3 µl of adapter-ligated library as template, with the following final concentrations: 1× AccuPrime reaction mix, 0.3 µM P7-P5 indexing primer mix, 7 U AccuPrime Pfx (a high fidelity polymerase, Thermo Scientific), and the following cycling protocol: 95 °C for 2 min, 12 cycles at 95 °C for 30 s, 55 °C for 30 s, 72 °C for 1 min, and a final extension at 72 °C for 5 min. Purification and size selection of the amplified and indexed library were then performed using Agencourt AMPure XP beads (Beckman Coulter, Brea, CA, USA), first using 0.5X bead: DNA ratio and secondly 1.8X to remove long and short (i.e., adapter dimers) fragments, respectively. Library concentration was measured with a high-sensitivity DNA chip on a Bioanalyzer 2100 (Agilent, Santa

Clara, CA, USA). Finally, the library was sequenced on Illumina HiSeq X flowcells with a 2×150 bp setup, incl. Xp kit (validated method) at the Science for Life Laboratory (National Genomics Institute, Stockholm). In total, we obtained 152 Gb data for the Casa del Diablo vulture. Extractions and library preparation were conducted in a separate aDNA lab (where no vulture tissue or DNA had ever been handled before) and appropriate precautions were taken to minimize the risk of contamination of the ancient sample (21).

After sequencing we took precautions against the influence from erroneous DNA degradation patterns. As these almost exclusively appear at the ends of sequence reads (22), we arbitrarily shortened all reads obtained from the museum study skins by deleting 5bp from both ends in order to reduce this “noise”. An analysis of the remaining damage pattern with mapDamage (23) shows that this procedure efficiently reduced the remaining DNA damage (Supplementary Figure 4).

Supplementary Methods 2. Estimating the divergence time between atratus *and* occidentalis*.*

We estimated the timing of the split between *occidentalis* and *atratus* using three different methods. First we translated the genetic distance observed between *occidentalis* and its nearest *atratus* relative (CA73 collected in Chile) in cytochrome *b* into years by using a mean average rate of divergence between two taxa of 2.1% per million years (24). We did this also for the distance observed between *occidentalis* and CA73 for the concatenation of nuclear introns (497 kb) using a mean average rate of divergence between two taxa of 0.128% per million years (25). Our third method to date the split between *occidentalis* and *atratus* was to use hPSMC (26) to estimate the time of the end of gene flow between them. We first generated haploid consensus genomes for the fossil and for CA73 using Samtools (27). The two haploid fasta files generated were then combined into a diploid, “hybrid” sequence using the hPSMC tool psmcfa_from_2_fastas.py. After this we ran the psmcfa output through PSMC (28) with the parameters (-p) "4+30*2+4+6+10" (29), number of iterations = 30, maximum 2N0 coalescent time = 15, initial theta/rho ratio = 5. We used psmc_plot.pl to translate this information into a plot and we assumed a mutation rate of 1.4 x 10^{-9} per base pair per generation (Zhang et al. 2014) and 14.2 years generation time (30).

The results are largely congruent. The estimated date for the split using mtDNA is 379 kya, and the corresponding date using nuclear introns is 329 kya (Supplementary Table 4). The PSMC curves for the two hybrid genomes indicate that the cessation of gene flow (i.e. when the effective population appears to be almost infinitely large) between the ancestors of the Peruvian *occidentalis* fossil and the phylogeographically closest *atratus* sample (Chile) occurred around some time before 300 kya, while the split between the ancestors of *occidentalis* and the population in U.S.A. is estimated to have occurred ca. 100 kya earlier (Fig. 3a).

Supplementary Table 1. Radiocarbon dates from Casa del Diablo. The data for mammals are from Villavicencio & Werdelin (2). ND = no data.

Collection number	Taxon	Element	^{14}C Lab number	Carbon (mg)	^{14}C age	Cal mean (cal yr BP)	Cal 2σ range (cal yr BP)	C:N bone collagen
A314	[*Coragyps*] *occidentalis*	right femur	Ua-70537	500	12098±44	13990	14100-13880	3.3
M1910	*Hippidion* cf. *devillei*	left hemimandible	CAMS 175037	1090	10980±90	12830	12700-13020	3.1
M1917c	*Hippidion* cf. *devillei*	right hemimandible	CAMS 175035	1090	11740±100	13530	13330-13740	3
M4103	*Hippidion* cf. *devillei*	right III metacarpal	CAMS 175039	840	11980±100	13780	13550-14060	3
M4093	*Hippidion* cf. *devillei*	mandible	CAMS 175038	830	11980±100	13780	13550-14060	3.07
M4099	*Hippidion* cf. *devillei*	right hemimandible	CAMS 175734	830	12400±80	14410	14070-14860	3.05
M4098	*Hippidion* cf. *devillei*	right hemimandible	CAMS 175036	950	12480±110	14580	14140-15070	3.08
M4200	*Vicugna vicugna*	right femur	CAMS 175750	960	12780±90	15170	14770-15510	ND
M1937a	*Lama guanicoe*	left femur	CAMS 175751	970	13260±90	15880	15580-16180	ND
M4445	*Catonyx* sp.	right hemimandible	CAMS 175745	1230	13690±100	16460	16150-16840	2.97
M4286	*Diabolotherium nordenskioldi*	right humerus	CAMS 175034	920	16300±180	19620	19150-20080	3
M5202	*Punomys lemminus*	right hemimandible	CAMS 175752	840	16870±150	20300	19920-20680	ND
M4290	*Megatherium* sp.	left astragalus	CAMS 175033	810	19180±260	23060	22470-23660	3

Supplementary Table 2. List of fossil localities with fossils referred to *Coragyps*. The first column gives the taxon to which the fossils are referred herein (justifications for changes of taxon names are given in the footnotes). The second column gives the taxon name given in the original publication or in the database of the museum where it is stored. An asterisk in the column for the elevation of the locality denotes that it has been inferred from GoogleEarth. The maximum and minimum ages are inferred from the age of the geological period given in the original publication or database record.

Taxon (inferred)	Taxon	Country	Province	Locality	lat	long	m asl		max age (inferred)	min age (inferred)	Ref.
atratus	*atratus*	Argentina	Buenos Aires	Pampean Region, Quequén Grande River, Paso Otero	-38.20	-59.12	96	Upper Pleistocene	0.0401	0.0355	74
atratus	cf. *atratus*	Ecuador	Santa Elena	La Libertad, La Carolina	-2.22	-80.88	25*	Upper Pleistocene	0.126	0.0117	34
atratus	*atratus*	Venezuela	Zulia	Mene de Inciarte	10.78	-72.23	115	Upper Pleistocene	0.126	0.0117	35
atratus	*atratus*	Mexico	Chiapas	Paso de la Amada	14.83	-92.50	13*	Holocene	0.0117	0	36
atratus	*atratus*	Peru	Piura	Talara, Talara Tar Seeps	-4.55	-81.12	85	Upper Pleistocene	0.014	0.014	11
atratus	*atratus*	USA	Florida	Alachua Co., Arredondo, 2A	29.60	-82.40	26*	late Rancholabrean	0.13	0.01	FLMNH
atratus[4)]	*atratus*	USA	Florida	Alachua Co., Haile, 11B	29.80	-82.10	45*	late Rancholabrean	0.13	0.01	37
atratus	*atratus*	USA	Florida	Alachua Co., Haile, 16A	29.68	-82.57	25*	late early Irvingtonian	1.6	1	12
atratus	*atratus*	USA	Florida	Alachua Co., Haile, 8A	29.70	-82.58	31*	Upper Pleistocene	0.129	0.1	FLMNH
atratus	*atratus*	USA	Florida	Citrus Co., Inglis, 1A	29.01	-82.67	10*	late Blancan - early Irvingtonian	1.9	1.6	12
atratus	*atratus*	USA	Florida	Citrus Co., Sabertooth Cave	28.86	-82.49	14*	late Rancholabrean	0.045	0.011	38
atratus	*atratus*	USA	Florida	Columbia Co., Ichetucknee River	29.96	-82.79	30*	late Rancholabrean	0.05	0.011	39
atratus[12)]	sp.	USA	Florida	DeSoto Co., DeSoto Shell Pit 5	27.00	-81.80	11*	early Irvingtonian	2	1.6	FLMNH
atratus[8)]	*atratus*	USA	Florida	Indian River Co., Vero Canal Site	27.65	-80.40	2*	late Rancholabrean	0.02	0.01	40

atratus	*atratus*	USA	Florida	Levy Co., Devil's Den	29.40	-82.50	25*	Upper Pleistocene-Holocene	0.02	0.01	FLMNH
atratus	*atratus*	USA	Florida	Levy Co., McLeod Limerock Mine Pocket A	29.40	-82.60	22*	middle Irvingtonian	0.8	0.6	12
atratus	*atratus*	USA	Florida	Miami-Dade Co., Cutler Hammock	25.36	-80.18	2*	late Rancholabrean	0.044	0.01	12
atratus	*atratus*	USA	Florida	Miami-Dade Co., Perrine 7.5' Quadrangle, NE1/4 of NW1/4 of section 35, T.55S., R.40E	25.70	-80.40	2*	Upper Pleistocene	0.126	0.0117	41
atratus	*atratus*	USA	Florida	Orange Co., Apopka, Rock Springs	28.72	-81.45	20*	late Rancholabrean	0.13	0.01	42
atratus	*atratus*	USA	Florida	Pinellas Co., Seminole Field	27.80	-82.70	10*	Upper Pleistocene	0.025	0.0117	38
atratus	*atratus*	USA	Florida	Sarasota Co., Little Salt Spring	27.10	-82.20	10*	Upper Pleistocene	0.126	0.012	FLMNH
atratus [9)]	*occidentalis*	USA	Florida	Sumter Co., Coleman, 2A	28.76	-82.05	20*	late Irvingtonian	0.6	0.3	13, 12
atratus	*atratus*	USA	Georgia	Bartow Co., Kingston Salpeter Cave	34.21	-84.92	283*	Upper Pleistocene	0.126	0.0117	43
atratus	*atratus*	USA	Maryland	Allegany Co., Cumberland Bone Cave	39.69	-78.79	245	Calabrian-Chibanian	0.84	0.66	14
occidentalis	*occidentalis*	Peru	Puno	Tirapata, Casa del Diablo Cave	-15.27	-70.53	3819	Upper Pleistocene	0.023	0.0128	1, 2
occidentalis	*occidentalis*	Mexico	Chihuahua	Cueva Jiménez	27.03	-104.82	1450	Mid Wisconsin-Holocene	0.055	0.012	44
occidentalis	*occidentalis*	Mexico	Nuevo León	Zaragoza, Cueva de San Josecito	23.96	-99.91	2250	Upper Pleistocene	0.126	0.012	45, 10, 46
occidentalis	*occidentalis*	USA	Arizona	Mohave Co., Rampart Cave	36.10	-113.93	534	Mid/Late Wisconsin	0.04	0.0117	48
occidentalis	*occidentalis*	USA	California	Eldorado Co., Hawver Cave	38.91	-121.03	410*	Upper Pleistocene	0.126	0.012	UCMP
occidentalis	*occidentalis*	USA	California	Kern Co., McKittrick asphalt deposit	35.30	-118.50	920*	Mid/Late Wisconsin	0.055	0.0117	50, 51
occidentalis	*occidentalis*	USA	California	Los Angeles Co., Rancho La Brea	34.05	-118.24	109*	Upper Pleistocene	0.044	0.004	52
occidentalis	*occidentalis*	USA	California	Santa Barbara Co., Carpinteria asphalt deposit	34.41	-119.52	135*	Upper Pleistocene	0.126	0.0117	54, 55
occidentalis	*occidentalis*	USA	California	Shasta Co., Mt. Diablo Meridian, Potter Creek Cave	40.70	-122.30	270*	Upper Pleistocene	0.126	0.011	50
occidentalis	*occidentalis*	USA	California	Shasta Co., Samwell Cave	40.90	-122.20	750*	Upper Pleistocene	0.126	0.012	UCMP

occidentalis	*occidentalis*	USA	Nevada	White Pine Co., Smith Creek Cave	38.80	-114.20	1890	Upper Pleistocene	0.126	0.011	65
occidentalis	*occidentalis*	USA	New Mexico	Bernalillo Co., Isleta Cave No. 1	34.90	-106.89	1716	Late Wisconsin	0.025	0.0117	76
occidentalis	*occidentalis*	USA	New Mexico	Doña Ana Co., Conkling Cavern	33.00	-106.50	1299	Late Wisconsin	0.025	0.0117	56
occidentalis	*occidentalis*	USA	New Mexico	Eddy Co., Carlsbad, Dry Cave (older strata)	32.37	-104.45	1280	Chibanian	0.33	0.25	60, 61, 76
occidentalis	*occidentalis*	USA	New Mexico	Eddy Co., Carlsbad, Dry Cave (younger strata)	32.37	-104.45	1280	Early Wisconsin/ Holocene	0.08	0.01	76, 61
occidentalis	*occidentalis*	USA	New Mexico	Grant Co., Howells Ridge Cave (=Hachita Cave)	31.85	-108.46	1675	Late Wisconsin/ Holocene	0.012	0.01	62
occidentalis	*occidentalis*	USA	New Mexico	Hidalgo Co., U-Bar Cave	31.47	-108.43	1540	Mid/Late Wisconsin	0.055	0.0117	76
occidentalis	*occidentalis*	USA	New Mexico	Otero Co., Rough Canyon, Pendejo Cave	32.42	-105.92	1490	Mid Wisconsin	0.055	0.025	63
occidentalis	*occidentalis*	USA	New Mexico	Sandoval Co., Las Huertas Canyon, Sandia Cave	35.25	-106.40	2280	Late Wisconsin	0.025	0.012	64
occidentalis [1)]	*atratus*	USA	New Mexico	Eddy Co., Carlsbad, Burnet Cave (=Rocky Arroyo Cave)	32.37	-104.78	1951	Late Wisconsin	0.025	0.0117	57, 58
occidentalis [11)]	*occidentalis*	USA	New Mexico	Eddy Co., Carlsbad, Dark Canyon Cave	32.30	-104.35	1100	Mid/Late Wisconsin	0.055	0.0117	59
occidentalis [2)]	cf. *atratus*	USA	New Mexico	Hidalgo Co., U-Bar Cave	31.47	-108.43	1540	Mid Wisconsin/ Holocene	0.055	0.01	76
occidentalis	*occidentalis*	USA	Oregon	Wasco Co., Fivemile Rapids	45.63	-121.11	85*	Holocene	0.009	0.009	77, 78
occidentalis	*occidentalis*	USA	Texas	Bexar Co., Friesenhahn Cave	29.00	-98.00	103*	Upper Pleistocene	0.019	0.017	66, 67
occidentalis	*occidentalis*	USA	Texas	Culberson Co., Lower Sloth Cave	31.43	-104.18	2000	Late Wisconsin	0.025	0.0117	68, 69
occidentalis	*occidentalis*	USA	Texas	Kerr Co., Hall's Cave	30.08	-99.32	600*	Upper Pleistocene	0.126	0.0117	66
occidentalis [10) 13)]	*occidentalis*	USA	Florida	Marion Co., Reddick, 1A, 1B, 1C	29.10	-82.30	29*	Rancholabrean	0.13	0.01	49
occidentalis [3)]	*occidentalis*	USA	Texas	Hudspeth Co., Sierra Diablo Cave, strata A and F	31.18	-105.07	1660	Mid/Late Wisconsin	0.055	0.0117	70
sp.	sp.	Peru	Huánuco	Jatun Uchco	-10.13	-76.20	2150	Upper Pleistocene	0.126	0.0117	71
sp. [14)]	*occidentalis*	Mexico	Sonora	El Golfo de Santa Clara	31.75	-114.55	46	Irvingtonian (Calabrian)	1.3	0.78	16, 15, 47

sp.	sp.	USA	California	Los Angeles Co., San Pedro Lumber Yard	34.00	-118.00	315*	Rancholabrean	0.025	0.012	51
sp. [5)]	*atratus*	USA	New Mexico	Bernalillo Co., Albuquerque, Edith Fm.	35.08	-106.60	1600*	Pleistocene	0.126	0.0117	75
sp. [7)]	*atratus*	USA	Nevada	Clark Co., Devil Peak	35.72	-115.43	1098	Mid Wisconsin	0.055	0.025	72
sp. [6)]	*atratus?*	USA	Texas	Culberson Co., Dust Cave	31.42	-104.20	1980	Late Wisconsin	0.025	0.0117	73

NOTES: [1)] Howard (1968) questions this is *atratus* (based on size). [2)] Arthur Harris in mail 2021-02-10: "The U-Bar specimen is a "cf." with the comment "QUALITATIVE CHARACTERS NEARER *CORAGYPS* THAN *CATHARTES*", which suggests to me that the latter is a possibility." [3)] Arthur Harris in mail 2021-02-10 concerning the bone in stratum A: "I have the Sierra Diablo Cave fifth of a radius listed as *C. coragyps*[sic, presumambly he means *occidentalis*]. However, stratum A is disturbed with some definite Pleistocene elements present. Apparently at the time of identification, I indicated that my subjective opinion was that it was Pleistocene." [4)] Originally I had this as *occidentalis* but I am not sure from where I got this. It is recorded as *atratus* in GBIF and in the museum's database. [5)] Arthur Harris in mail 2021-02-10 concerning the bone in stratum A: "I think Brodkorb identified the Burnet Cave and Albuquerque specimens, but I doubt he considered *coragyps*[sic, from rest of his mail it is clear he meant *occidentalis*"] [6)] UTEP website: "Santucci et al. (2001) stated that *C. atratus* is documented from Dust Cave, but gave no indication that the *atratus/occidentalis* situation was considered." [7)] UTEP website: "Reynolds, Reynolds, and Bell (1991) listed without comment *C. atratus* from Devil Peak." [8)] No *Coragyps* is listed in the FMNH database - only one bone of *Cathartes aura*. Possibly it has been re-identified. [9)] Ritchie (1980) lists them as *occidentalis* after finding they agree in size with some specimens from Reddick (but size seems to agree better with *atratus*!). The FMNH database lists these specimens as *atratus*. Emslie (1998) also lists them as *atratus*. [10)] Gut & Ray (1963) list *occidentalis* from "Reddick". The FMNH database lists 41 specimens as *atratus* and 14 specimens as *Coragyps* sp. from Reddick 1A, 1B and 1C. [11)] Howard (1971) writes: "The *Gymnogyps* and *Coragyps* bones are assigned to the Pleistocene species on the basis of their size." [12)] Steve Emslie in mail 2021-05-12: "Only a distal left radius (UF 232087) labeled as cf. *Coragyps*. It compares well with modern *Coragyps atratus* in size and characters and measures breadth, 13.0 mm, depth 7.0 mm." [13)] Steve Emslie in mail 2021-05-12: "These bones include complete and partial tarsometatarsi, coracoids, ulnae, tibiotarsi, and one femur. All had measurements that tended to be larger than Howard's measurements for *atratus* and for the most part fit with *occidentalis*, especially the southern subspecies from San Josecito Cave." 14) a singel distal right humerus of *Coragyps* has been collected in El Golfo (Fred W Croxen in email 2021-08-21). It is listed as *Coragyps occidentalis*in Croxen et al. (2007) based on identifications by Steadman. Steadman writes (email 2021-08-18) that he measured the distal width to 26.2 mm, while Emslie twice has measured it to 25.6 mm (emails 2021-08-18 and 2022-03-18). Emslie's measurement falls within the size range for *atratus* (Howard 1968), but is also within the size range of Mexican *occidentalis* collected in San Josecito. Given the exceptionally old age of this specimen and its intermediate size between *occidentalis* and recent *atratus*, we question its assignment to occidentalis and regard it as *Coragyps* sp..

Supplementary Table 3. Skeletal measurements for *Coragyps atratus* and *occidentalis*. Cranial data from Fisher (7) and postcranial data from Howard (8). Measurements are also given for the Pleistocene *Coragyps* collected in Casa del Diablo, Peru.

	atratus			*occidentalis* (RLB)			Peru specimen
	mean	min	max	mean	min	max	
Length of cranium	51.9	51.3	52.7	54.4	52.8	56.5	
Height of cranium	32.1	30.5	33.7	34.5	30.2	35.2	
Postorbital width	36.2	35.3	38.4	39.5	36.2	40.7	
Temporal width	33.4	33	35.1	36.1	33.2	37.9	
Width of hinge	21.9	21.5	22.3	24.1	22.8	26	
Length premaxillary	63.8	62.4	65.4	70	68.4	72.4	
Length premaxillary anterior to nares	21.7	20.5	22.8	24.5	23	25.7	
Width of bill	13.4	12.3	14	15.2	14.2	16.3	
Depth of bill	7.4	6.7	8.2	7.9	7.2	8.7	
Length nares	25.9	24.8	27.3	31.2	29.2	33	
Opisthotic width	32.6	31.7	33.2	32.9	29	35.1	
Occipital width	16.2	15	16.6	17.4	15.3	18.5	
Coracoid length	58.9	55.6	60.3	66.2	63.9	67.5	65.6
Coracoid shaft breadth	10.7	10.3	11.4	12.3	11.3	13.3	12.3
Humerus length	135.4	124.8	142.2	148.5	142.6	155.9	
Humerus proximal breadth	26.4	24.7	27.9	30.6	28.5	32.4	
Humerus distal breadth	24.2	22.5	25.7	27.4	26.1	29.1	
Ulna length	155.9	144.3	161.5	166.2	156.3	179.3	172
Ulna proximal breadth	15.8	14.2	17	17	16	18.2	
Cmc length	74.9	67.5	78.2	81.8	74.1	87.6	83.5
Cmc proximal breadth	8.6	8.3	9.3	9.7	9.1	10.7	9.6

Femur length	84.7	79.2	87.9	95	88.8	102.2
Femur distal breadth	20.3	19	21	23.4	21.1	24.3
Tibiotarsus length	140.3	133.2	146.1	145.4	138	154.7
Tibiotarsus distal breadth	12.7	12.4	13.2	14.4	13.4	15.9
Tmt length	83	76	86	82.8	78.3	88.8
Tmt proximal breadth	14.9	14.3	15.8	16.9	16	17.9
Tmt distal breadth	16.4	15.4	17.7	17.5	16.5	18.7

Supplementary Table 4. Molecular dating of the split between *occidentalis* fossil and the closest *atratus* sample (from Chile) in the population-level phylogeny (Fig. 2c). Mutation rates used are from (24) for mtDNA and (25) for nuDNA.

	Observed p-distance	Estimated time of divergence
mtDNA (cytochrome *b)*	0.80%	378 597 kya
Nuclear introns (485 kb)	0.42%	328 717 kya

Supplementary Table 5. Samples used in the molecular analyses.

Specimen no.	Tissue	Extraction	Locality	Date collected	Sex	Mapping coverage	Phylo-geography	PCA
USNM 105459	Study skin	CA03	United States, Texas	10 Jan 1885	Male	1.9	x	x
USNM 150071	Study skin	CA04	United States, Florida	14 Mar 1895	Male	2.4	x	x
USNM 152126	Study skin	CA06	United States, Florida	12 Feb 1896	Male	2.1	x	x
USNM 175429	Study skin	CA07	United States, Florida	24 Feb 1901	Female	2.0	x	x
USNM 176012	Study skin	CA10	United States, Florida	16 Mar 1895	Male	3.0	x	x
USNM 185275	Study skin	CA11	Mexico, Michoacan De Ocampo	13 Mar 1903	Male	2.5	x	x
USNM 185276	Study skin	CA12	Mexico, Michoacan De Ocampo	16 Mar 1903	Male	1.9	x	x
USNM 185278	Study skin	CA13	Mexico, Michoacan De Ocampo	18 Mar 1903	Unknown	3.1	x	x
USNM 230141	Study skin	CA14	Panama	29 May 1911	Male	0.4		x
USNM 232608	Study skin	CA15	Panama, Canal Zone	8 Feb 1912	Female	5.7	x	x
USNM 232614	Study skin	CA19	Panama, Canal Zone	12 Feb 1912	Female	1.7		x
USNM 299197	Study skin	CA21	United States, Virginia, Fairfax City, Fairfax	27 Jan 1927	Male	4.8	x	x
USNM 299198	Study skin	CA22	United States, Florida, Lafayette, Mayo	14 Jun 1926	Female	1.3	x	x
USNM 339175	Study skin	CA23	United States, South Carolina, Anderson, Anderson	2 Mar 1931	Male	3.5	x	x
USNM 352246	Study skin	CA24	United States, Tennessee	12 Oct 1937	Male	5.9	x	x
USNM 356694	Study skin	CA25	USA, Kentucky	31 May 1938	Female	0.5		x
USNM 362970	Study skin	CA26	United States, South Carolina	1 Oct 1940	Male	3.8	x	x
USNM 368105	Study skin	CA27	Brazil, Distrito Federal	19 Nov 1940	Female	4.5	x	x
USNM 368475	Study skin	CA29	Colombia, La Guajira, Carraipia	23 Jun 1941	Female	2.5	x	x
USNM 370549	Study skin	CA30	Mexico, Veracruz-Llave	15 Mar 1901	Male	3.5	x	x
USNM 370550	Study skin	CA31	Mexico, Veracruz-Llave	Unknown	Unknown	0.4		x
USNM 370551	Study skin	CA32	Mexico, Veracruz-Llave	22 Apr 1901	Male	4.1	x	x
USNM 379039	Study skin	CA33	United States, Georgia	26 Jul 1939	Male	2.6	x	x
USNM 388833	Study skin	CA36	Colombia, Cauca, Popayan	22 Nov 1945	Male	1.9	x	x
USNM 390683	Study skin	CA37	Paraguay	20 Jan 1940	Female	1.7	x	x
USNM 483165	Study skin	CA39	Mexico, Veracruz-Llave	9 Apr 1963	Male	6.0	x	x
USNM 513817	Study skin	CA40	Brazil, Para	29 Jul 1964	Male	6.4	x	x
USNM 513818	Study skin	CA41	Brazil, Para	29 Jul 1964	Male	2.8		x
USNM 513819	Study skin	CA42	Brazil, Para	29 Jul 1964	Male	8.0	x	x
USNM 565379	Study skin	CA43	United States, Indiana, Franklin, Bath	22 Feb 1887	Unknown	1.9	x	x

AMNH 45070	Study skin	CA49	USA, South Carolina	1889	Unknown	0.7		x
AMNH 60664	Study skin	CA50	Trinidad and Tobago, Caura	13 Apr 1894	Female	3.5	x	x
AMNH 80632	Study skin	CA51	USA, Texas, Corpus Christi	Mar. 1877	Female	0.8		x
AMNH 97888	Study skin	CA52	Colombia, Bonda	24 Feb 2021	Male	1.1	x	x
AMNH 104595	Study skin	CA53	Mexico, Nayarit, Arroyo de Platinar, nr. A. de Cañas, S. Tepic	9 Mar 1905	Female	0.3		x
AMNH 113434	Study skin	CA54	Chile, Araucanía, Cautin, Temuco, Maquehue	5 Sep 1907	Male	0.6		x
AMNH 113435	Study skin	CA55	Chile, Araucanía, Cautin, Temuco, Maquehue	10 May 1907	Female	1.1		x
AMNH 123927	Study skin	CA56	Ecuador, Quito	23 Jul 1913	Male	3.4	x	x
AMNH 123928	Study skin	CA57	Ecuador, Quito	23 Jul 1913	Male	7.3	x	x
AMNH 132966	Study skin	CA58	Colombia, Antioquia, Puerto Valdivia, Cauca R.	26 Dec 1914	Male	4.8	x	x
AMNH 147901	Study skin	CA59	Argentina, Mendoza, TunuyÃ¡n	1 Nov 1910	Female	1.6	x	x
AMNH 165520	Study skin	CA60	Chile, Los Ríos, Corral	6 Oct 1913	Male	0.6		x
AMNH 186287	Study skin	CA62	Peru, Pt. Pariñas	24 Jan 1925	Female	0.9	x	x
AMNH 186472	Study skin	CA63	Chile, Araucanía, Angol	7 Jun 1924	Male	1.0		x
AMNH 241495	Study skin	CA64	Brazil, Maranhão, São José	28 Jan 1926	Female	1.3	x	x
AMNH 352004	Study skin	CA68	USA, North Carolina, Raleigh	1 Feb 1894	Female	0.9		x
AMNH 407936	Study skin	CA69	Panama, Pacheca I., Pearl Is.	10 Feb 1941	Female	4.6	x	x
AMNH 446853	Study skin	CA70	Panama, Gigante Bay, Gatun Lake	24 Jan 1937	Female	1.4	x	x
AMNH 460057	Study skin	CA71	Colombia, Meta, Rio Duda, Mt. Macarena	5 Feb 1942	Male	2.6		x
AMNH 469908	Study skin	CA72	Chile, Cabrero, Concepcion	4 Jul 1904	Male	1.0	x	x
AMNH 469909	Study skin	CA73	Chile, Penco - near Concepcion	7 Jul 1904	Female	2.0	x	x
AMNH 469910	Study skin	CA74	Chile, Valdivia	Sept 1897	Female	1.1	x	x
AMNH 469911	Study skin	CA75	Chile, Valdivia	11 Mar 1905	Male	1.8	x	x
AMNH 469931	Study skin	CA77	Mexico, Jalisco, Guadalajara	30 Mar 1892	Male	2.2		x
AMNH 469932	Study skin	CA78	Colombia, Antioquia, Medellin	Unknown	Unknown	1.7		x
AMNH 792356	Study skin	CA83	Argentina, Misiones	21 Jun 1961	Female	3.2	x	x
AMNH 798644	Study skin	CA85	Brazil, Goias, Annapolis	29 Dec 1936	Female	0.2		x
AMNH 798645	Study skin	CA86	Brazil, Mato Grosso, Maracaju	3 Jul 1937	Male	0.6		x
AMNH 798646	Study skin	CA87	Brazil, Mato Grosso, Maracaju	15 May 1937	Male	1.2	x	x
AMNH 798647	Study skin	CA88	Brazil, Goias, Annapolis	4 Feb 1937	Male	0.3		x
AMNH 798648	Study skin	CA89	Brazil, Mato Grosso, Maracaju	22 Jul 1937	Female	1.0		x
AMNH 798654	Study skin	CA91	Brazil, Goias, Annapolis	24 Feb 1937	Male	0.5		x
NRM 947124	Muscle	de novo	Paraguay, Alto Paraguay, Rio Negro	15 Sept. 1994	Male		x	
NRM PAL-PZ A312-315	Subfossil bone		Casa del Diablo, Altiplano, Peru		Unknown	1.2	x	x

Supplementary Table 6. Statistics for the three phylogenomic data sets used.

	Genus-level relationships	Population-level phylogeny of *Coragyps*	To root the population-level phylogeny
No. taxa in alignment	13	43	13
No. exon alignments	892	–	518
Mean alignment length	567	–	690
Mean proportion parsimony-informative	4.80%	–	0.55%
No. intron alignments	–	1 179	3 633
Mean alignment length	–	497	1 679
Mean proportion parsimony-informative	–	1.40%	0.32%
No. of parsimony-informative characters used in the Bayesian analysis	24 052	6 424	21 083
Alignment gaps and missing data	31%	15%	39%

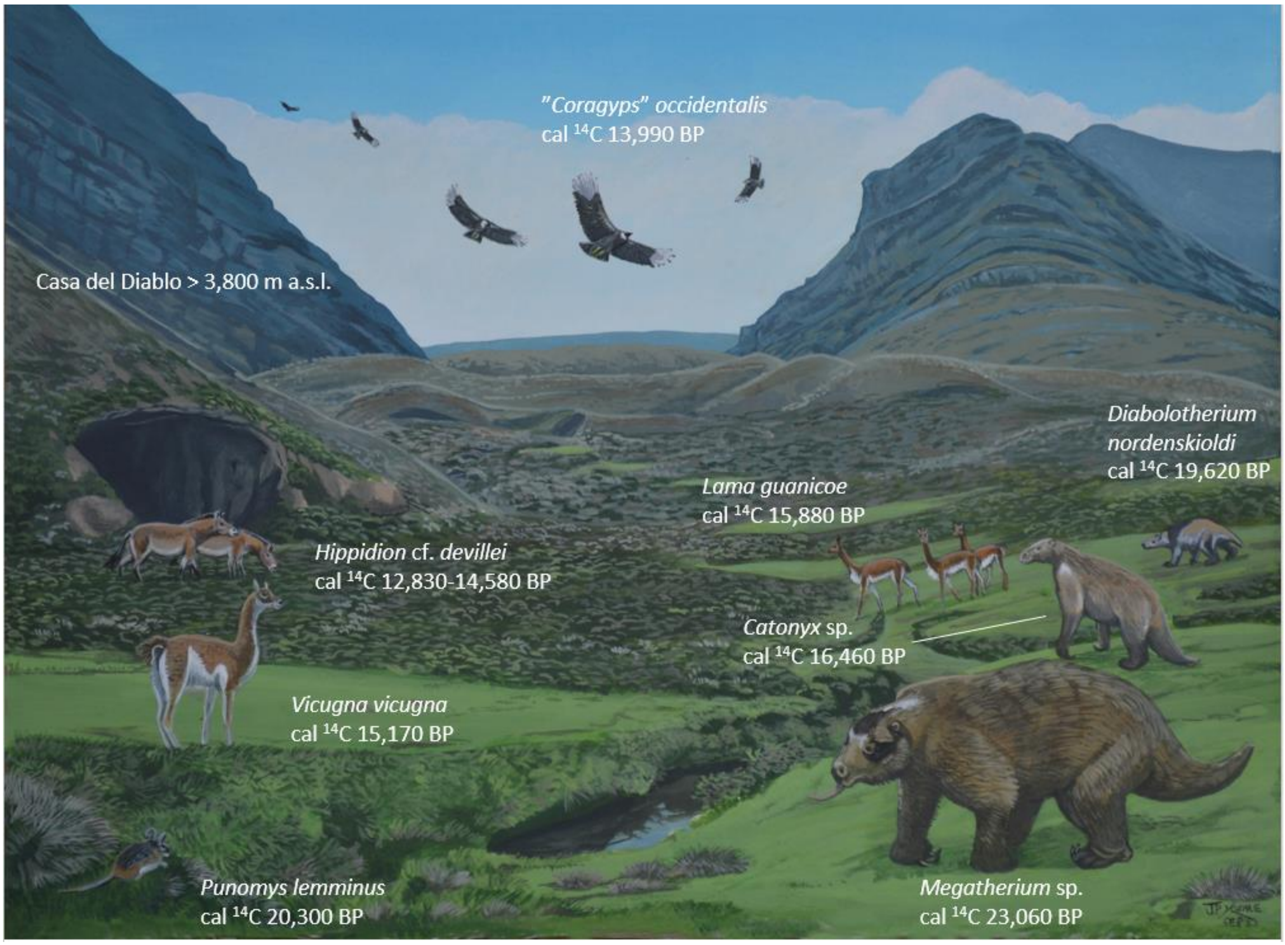

Supplementary Figure 1. Reconstruction of the Late Pleistocene environment in the Altiplano of southern Peru. Several fossils of mammals and birds have been collected in the cave Casa del Diablo situated at c. 3,800 m a.s.l. Included in this reconstruction are the species collected in the cave that have been ^{14}C dated. Original painting by Julian P. Hume.

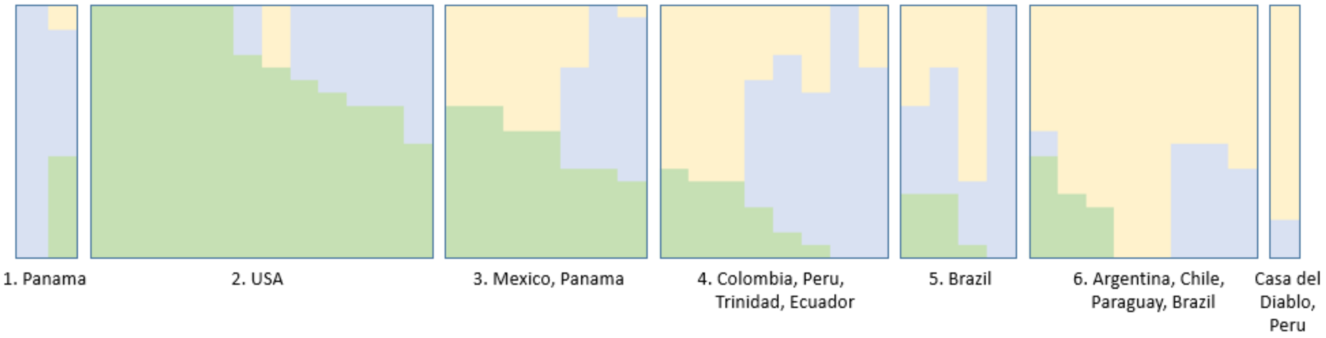

Supplementary Figure 2. Estimates of individual admixture proportions using NGSadmix. The number of clusters tested ranged from K = 1 to 8 and their fit to the data was evaluated using the Evanno method (79). K=3 (for which the results are shown) was found optimal to explain the variation in the data set. One bar refers to a single individual's proportional ancestry. The groupings of individuals are made according to how they fall in the phylogenomic analysis (numbers refer to Figure 2c).

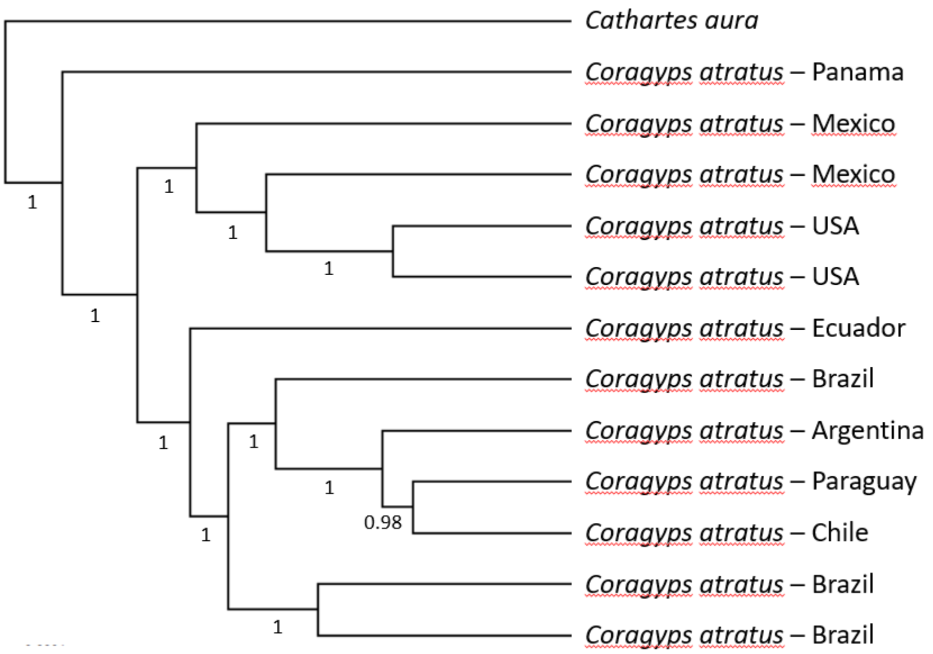

Supplementary Figure 3. Phylogenomic analysis of 15 individuals of *Coragyps atratus* and with *Cathartes aura* as outgroup based on 518 exonic and 3,633 intronic alignments. The numbers at the nodes indicate posterior probabilities. Based on this result we rooted the population-level phylogeny of *Coragyps atratus* (Fig. 2c) using the same individual from Panama as included here.

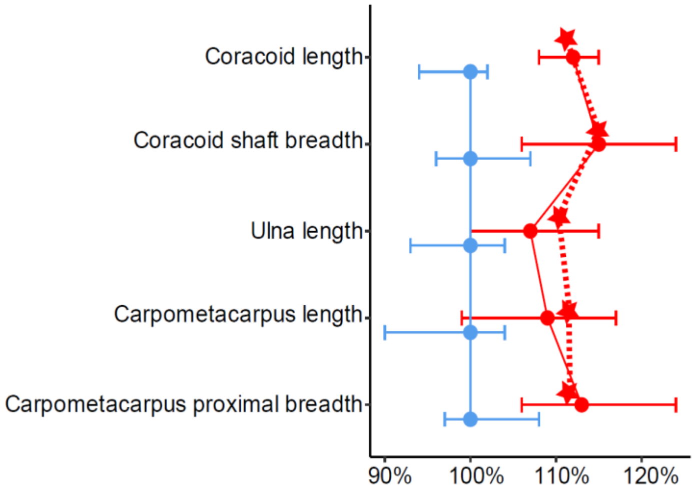

Supplementary Figure 4. The *occidentalis* specimen from Casa del Diablo agrees well with the proportions of *occidentalis* in North America. The body proportions of the Casa del Diablo specimens (marked with red stars) differ from *Coragyps atratus* (blue dots), but are fairly similar to the average for the La Brea specimens of *occidentalis* (red dots).

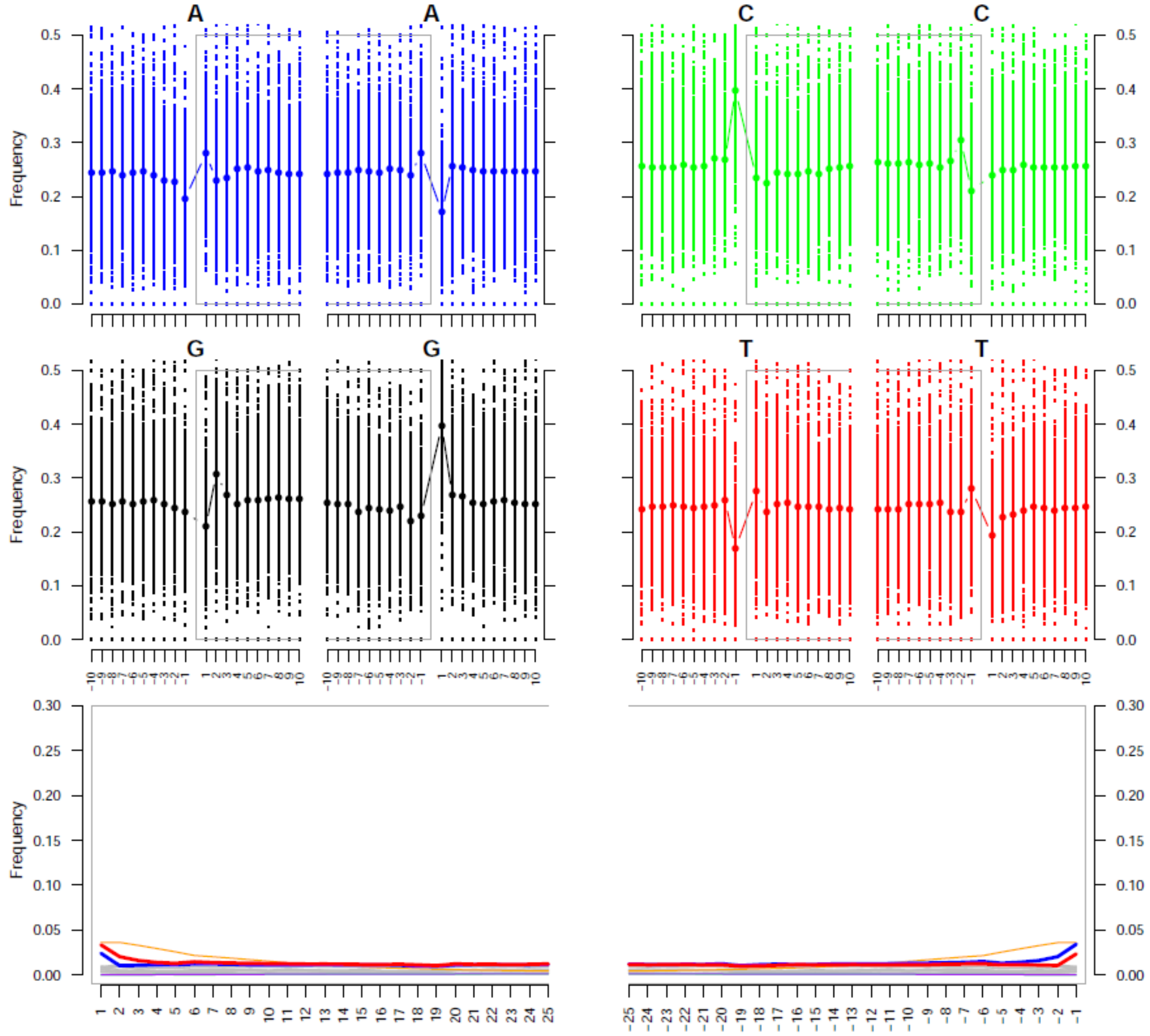

Supplementary Figure 5. Analysis using MapDamage of aDNA fragmentation and misincorporation patterns of sequencing read data from the fossil *occidentalis*.

Supplementary References

1. Nordenskiöld E. Ein neuer fundort für Säugetierfossilien in Peru. Ark. Zool. 1908; 4:13-21.
2. Villavicencio NA, Werdelin L. The Casa del Diablo cave (Puno, Peru) and the late Pleistocene demise of megafauna in the Andean Altiplano. Quaternary Science Reviews 2018; 195:21-31.
3. Jarvis ED, Mirarab S, Aberer AJ, Li B, Houde P, Li C, Ho SYW, et al. Whole-genome analyses resolve early branches in the tree of life of modern birds. Science. 2014; 346:1320-1331.
4. Ericson PGP, Anderson CL, Britton T, Elzanowski A, Johansson US, M. Källersjö M, et al. Diversification of Neoaves: integration of molecular sequence data and fossils. Biology Letters. 2006; 2:543-547.
5. Hackett SJ, Kimball RT, Reddy S, Bowie RCK, Braun EL, Braun MJ, et al. A phylogenomic study of birds reveals their evolutionary history. Science. 2008; 320:1763-1768.
6. Prum RO, Berv J, Dornburg A, Field DJ, Townsend JP, Moriarty Lemmon E, et al. A comprehensive phylogeny of birds (Aves) using targeted next-generation DNA sequencing. Nature. 2015; 526:569-573.
7. Fisher HL. The skulls of the cathartid vultures. The Condor. 1944; 46:272-296.
8. Howard H. Limb measurements of the extinct vulture, Coragyps occidentalis. Papers of the Archaeological Society of New Mexico. 1968; 1:115-127.
9. Simpson GG. Tempo and Mode in Evolution. New York: Columbia Univ. Press; 1944.
10. Steadman DW, Arroyo-Cabrales L, Johnson E, Fabiola Guzman A. New Information on the Late Pleistocene birds from San Josecito Cave, Nuevo León, Mexico. The Condor. 1994; 96:577-589.
11. Campbell KE, The non-passerine Pleistocene avifauna of the Talara Tar Seeps, northwestern Peru. Royal Ontario Museum Life Sciences Contribution. 1979; 118:1-203.
12. Emslie SD, Avian community, climate, and sea-level changes in the Plio-Pleistocene of the Florida Peninsula. Ornithological Monographs. 1998; 50:1-113.
13. Ritchie TL, Two mid-Pleistocene avifaunas from Coleman, Florida. Bull. Fla. State Mus. Biol. Sci. 1980: 26:1-36.
14. James HF, The Irvingtonian avifauna of Cumberland Bone Cave, Maryland. Zootaxa. 2020; 4772:111-131.

15. Sussman DR, Croxen III FW, McDonald HG, Shaw CA, Fossil porcupine (Mammalia, Rodentia, Erethizontidae) from El Golfo de Santa Clara, Sonora, Mexico, with a review of the taxonomy of the North American erethizonitids. Contributions in Science. 2016; 524:1-29.
16. Croxen III FW, Shaw CA, Sussman DR. Pleistocene geology and paleontology of the Colorado River delta at Golfo de Santa Clara, Sonora, Mexico. In: Reynolds RE, editor. The 2007 Desert Symposium field guide and abstracts. San Diego, California: Sunbelt Publications; 2007. pp. 84-89.
17. Dabney J, Knapp M, Glocke I, Gansauge MT, Weihmann A, Nickel B, et al. Complete mitochondrial genome sequence of a Middle Pleistocene cave bear reconstructed from ultrashort DNA fragments. Proc. Natl. Acad. Sci. U.S.A. 2013; 110:15758-15763.
18. Meyer M, Kircher M. Illumina sequencing library preparation for highly multiplexed target capture and sequencing. Cold Spring Harb. Protoc. 2010; 5:pdb.prot5448.
19. Briggs AW, Stenzel U, Meyer M, Krause J, Kircher M, Pääbo S, Removal of deaminated cytosines and detection of in vivo methylation in ancient DNA. Nucleic Acids Res. 2010; 38:e87.
20. Kircher M, Sawyer S, Meyer M. Double indexing overcomes inaccuracies in multiplex sequencing on the Illumina platform. Nucleic Acids Res. 2012; 40:e3.
21. Knapp M, Stiller M, Meyer M. Generating barcoded libraries for multiplex high-throughput sequencing. In: Shapiro B, Hofreiter M, editors. Ancient DNA: Methods and Protocols, Methods in Molecular Biology. New York: Humana, Springer; 2012. pp. 155-170.
22. Schubert M, Ginolhac A, Lindgreen S, Thompson JF, Al-Rasheid KA, Willerslev E, et al. Improving ancient DNA read mapping against modern reference genomes. BMC Genomics. 2012; 13:178.
23. Ginolhac A, Rasmussen M, Gilbert MT, Willerslev E, Orlando L. mapDamage: testing for damage patterns in ancient DNA sequences. Bioinformatics. 2011; 27:2153-2155.
24. Lerner HR, Meyer M, James HF, Hofreiter M, Fleischer RC. Multilocus resolution of phylogeny and timescale in the extant adaptive radiation of Hawaiian honeycreepers. Curr. Biol. 2011; 21:1838-1844.
25. Ellegren H. Molecular evolutionary genomics of birds. Cytogenet. Genome Res. 2007; 117:120-130.

26. Cahill JA, Soares AER, Green RE, Shapiro B. Inferring species divergence times using pairwise sequential Markovian coalescent modelling and low-coverage genomic data. Phil. Trans. R. Soc. B. 2016; 371:20150138.
27. Li H, Handsaker B, Wysoker A, Fennell T, Ruan J, Homer N, et al. The Sequence Alignment/Map format and SAMtools. Bioinformatics. 2009; 25:2078-2079.
28. Li H, Durbin R. Inference of human population history from individual whole-genome sequences. Nature. 2011; 475:493-496.
29. Nadachowska-Brzyska K, Li C, Smeds L, Zhang G, Ellegren H. Temporal dynamics of avian populations during Pleistocene revealed by whole-genome sequences. Curr. Biol. 2015; 25:1375-1380.
30. Bird JP, Martin R, Akçakaya HR, Gilroy J, Burfield IJ, Garnett ST, et al. Generation lengths of the world's birds and their implications for extinction risk. Conservation Biology. 2020; 34:1252-1261.
31. García-Moreno J, Arctander P, Fjeldså J. A case of rapid diversification in in the Neotropics: phylogenetic relationships among *Cranioleuca* spinetails (Aves: Furnariidae). Mol. Phylogen. Evol. 1999; 12:273-281.
32. Kirk DA, Mossman MJ. Turkey Vulture (*Cathartes aura*), version 1.0. In: Poole AF, Gill FB, editors. Birds of the World.Ithaca, NY: Cornell Lab of Ornithology; 2020.
33. Brodkorb P. Geographical variation in the black vulture. Papers of the Michigan Academy of Science, Arts and Letters. 1944. 29:115-121.
34. Campbell KE. The late Pleistocene avifauna of La Carolina, southwestern Ecuador. Smithsonian Contributions to Paleobiology. 1976; 27:155-168.
35. Steadman DW, Oswald JA, Rincón AD. The diversity and biogeography of late Pleistocene birds from the lowland Neotropics. Quaternary Research. 2015; 83:555-564.
36. Steadman DW, Tellkamp MP, Wake TA. Prehistoric exploitation of birds on the Pacific coast of Chiapas, Mexico. The Condor. 2003; 105:572-579.
37. Ligon JD. A Pleistocene avifauna from Haile, Florida. Bulletin of the Florida State Museum, Biological Sciences. 1966; 10:127-158.
38. Wetmore A. The avifauna of the Pleistocene in Florida. Smithsonian Miscellaneous Collections. 1931; 85:1-41.

39. McCoy JJ. The fossil avifauna of Itchtucknee River, Florida. The Auk. 1963; 80:335-351.
40. Weigel RD. Fossil vertebrates of Vero, Florida. Florida Geological Survey, Special Publication. 1962; 10:1-59.
41. Emslie SD, Morgan GS. Taphonomy of a late Pleistocene carnivore den, Dade County, Florida. In: Steadman DW, Mead J, editors. Late Quaternary Environments and Deep History: A Tribute to Paul Martin. Hot Springs South Dakota; 1995. pp. 65-83.
42. Woolfenden GE. A Pleistocene avifauna from Rock Spring, Florida. Wilson Bulletin. 1959; 71:183-187.
43. Steadman DW. Late Pleistocene birds from Kingston Saltpeter Cave, southern Appalachian Mountains, Georgia. Bulletin of the Florida Museum of Natural History. 2005; 45:231-248.
44. Messing HJ. A Late Pleistocene-Holocene Fauna from Chihuahua, Mexico. The Southwestern Naturalist. 1986; 31:277-288.
45. Miller L. The Pleistocene birds of San Josecito Cavern, Mexico. Univ. California Publ. Zool. 1943; 47:143-168.
46. Arroyo-Cabrales J, Johnson E. Catálogo de los ejemplares tipo procedentes de la Cueva de San Josecito, Nuevo León, México. Revista Mexicana de Ciencias Geológicas. 2003; 20:79-93.
47. Shaw CA, Croxen III FW. Geology and paleontology of the early-middle Pleistocene El Golfo beds, Sonora, Mexico - A field guide. The Geological Society of America Field Guide. 2019; 55:499-517.
48. Carpenter MC. Late Pleistocene Aves, Chiroptera, Perissodactyla, and Artiodactyla from Rampart Cave, Grand Canyon, Arizona. M.Sc. Thesis, University of Northern Arizona, Flagstaff. 2003.
49. Gut HJ, Ray CE. The Pleistocene vertebrate fauna of Reddick, Florida. Quarterly Journal of the Florida Academy of Sciences. 1963; 26:315-328.
50. Miller L. A second avifauna from the McKittrick Pleistocene. The Condor. 1935; 37:72-77.
51. Jefferson GT. A catalogue of Late Quaternary vertebrates from California. Part one; nonmarine lower vertebrate and avian taxa. Natural History Museum of Los Angeles County Technical Reports. 1991; 5:1-60.
52. Stock C, Harris JM. Rancho la Brea: A record of Pleistocene life in California. Natural History Museum of Los Angeles County, Science Series. 1992; 37:1-113.

53. Miller L. Pleistocene Birds from the Carpinteria Asphalt of California. Univ. Calif. Publ., Bull. Dept. Geol. Sci. 1931; 20:361-374.
54. Guthrie DA. An updated catalogue of the birds from the Carpinteria Asphalt, Pleistocene of California. Bulletin of the Southern California Academy of Sciences. 2009; 108:52-62.
55. Miller LH, Avifauna of the Pleistocene cave deposits of California. Univ. Calif. Publ., Bull. Dept. Geol. Sci. 1911; 6:385-400.
56. Howard H, Miller AH. Bird remains from cave deposits in New Mexico. The Condor. 1933; 35:15-18.
57. Wetmore A. Additional records of birds from cavern deposits in New Mexico. The Condor. 1932; 34:141-142.
58. Schultz CB, Howard EB. The fauna of Burnet Cave, Guadalupe Mountains, New Mexico. Proceedings of the Academy of Natural Sciences of Philadelphia. 1935; 87:273-298.
59. Howard H. Quaternary avian remains from Dark Canyon Cave, New Mexico. The Condor. 1971; 73:237-240.
60. Harris AH. Reconstruction of mid Wisconsin environments in southern New Mexico. National Geographic Research. 1987; 3:142-151.
61. Harris AH. The New Mexican late Wisconsin—east versus west. National Geographic Research. 1989; 5:205-217.
62. Howard H. Bird remains from a prehistoric cave deposit in Grant County, New Mexico. The Condor. 1962; 64:241-242.
63. Harris AH. The Pleistocene vertebrate fauna from Pendejo Cave. In: MacNeish RS, Libby JG, editors. Pendejo Cave. Albuquerque: University of New Mexico Press; 2003. pp. 36-65.
64. Brasso RL, Emslie SD. Two new late Pleistocene avifaunas from New Mexico. The Condor. 2006; 108:721-730.
65. Howard H. The prehistoric avifauna of Smith Creek Cave, Nevada, with a description of a new gigantic raptor. Bulletin of the Southern California Academy of Sciences. 1952; 51:50-54.
66. Toomey III RS. Late Pleistocene and Holocene faunal and environmental changes at Hall's Cave, Kerr County, Texas. Ph.D. Thesis, University of Texas at Austin. 1993.

67. Casto SD. Extinct and extirpated birds of Texas. Bulletin of the Texas Ornithological Society. 2002; 35:17-32.
68. Logan LE. The paleoclimatic implications of the avian and mammalian faunas of Lower Sloth Cave, Guadalupe Mountains, Texas. M.Sc. Thesis, Texas Tech University. 1977.
69. Logan LE. Paleoecological implications of the mammalian fauna of Lower Sloth Cave, Guadalupe Mountains, Texas. National Speleological Society, Bulletin. 1983; 45:3-11.
70. Vasquez JJ. Preliminary archaeological investigations at the Sierra Diablo Cave site: Paleoindian and archaic occupations in Hudspeth County, Texas. M.Sc. Thesis, University of Texas at El Paso. 2010.
71. Shockey BJ, et al., New Pleistocene Cave Faunas of the Andes of Central Perú: Radiocarbon Ages and the Survival of Low Latitude, Pleistocene DNA. Palaeontologia Electronica. 2009; 12:1-15.
72. Reynolds RE, Reynolds RL, Bell CJ. The Devil Peak sloth. In: Reynolds RE, editor. Crossing the Borders: Quaternary Studies in Eastern California and Southwestern Nevada. MDQRC 1991 Special Publication, San Bernardino County Museum Association, Redlands, CA; 1991. pp. 115-116.
73. Santucci VL, Kenworthy J, Kerbo R. An inventory of paleontological resources associated with National Park Service caves. Geologic Resources Division Technical Report NPS/NRGRD/GRDTR-01/02. 2001.
74. Cenizo MM, Agnolin FL, Pomi LH. A New Pleistocene bird assemblage from the Southern Pampas (Buenos Aires, Argentina). Palaeogeography, Palaeoclimatology, Palaeoecology. 2015; 420:65-81.
75. Brodkorb P. Catalogue of fossil birds: Part 2 (Anseriformes through Galliformes). Bull. Florida State Mus., Biol. Sci. 1964; 8:195-335.
76. Harris AH. Quaternary vertebrates of New Mexico. New Mexico Museum of Natural History and Science Bulletin. 1993; 2:179-197.
77. Miller L. Bird remains from an Oregon Indian midden. The Condor. 1957; 59:59-63.
78. Hansel-Kuehn V. The Dalles roadcut (5-Mile Rapids) avifauna: Evidence for a cultural origin. M.Sc. Thesis, Washington State University, Pullman. 2003.

79. Evanno G, Regnaut S, Goudet J. Detecting the number of clusters of individuals using the software structure: a simulation study. Molecular Ecology. 2005; 14:2611-2620.
