# Supplementary material for: A 14,000-year-old genome sheds light on the evolution and extinction of a Pleistocene vulture
Source: Commun Biol. 2022 Aug 23;5:857. doi: 10.1038/s42003-022-03811-0 (PMC9399080; doi:10.1038/s42003-022-03811-0)
Supplement: Supplementary file 3 — Reporting Summary [file 42003_2022_3811_MOESM3_ESM.pdf]

nature portfolio

Corresponding author(s): Per Ericson
Yamhua Qu

Last updated by author(s): Jul 22, 2022

Reporting Summary

Nature Portfolio wishes to improve the reproducibility of the work that we publish. This form provides structure for consistency and transparency in reporting. For further information on Nature Portfolio policies, see our Editorial Policies and the Editorial Policy Checklist.

Statistics

For all statistical analyses, confirm that the following items are present in the figure legend, table legend, main text, or Methods section.

n/a	Confirmed	
☐	☒	The exact sample size (*n*) for each experimental group/condition, given as a discrete number and unit of measurement
☐	☒	A statement on whether measurements were taken from distinct samples or whether the same sample was measured repeatedly
☐	☒	The statistical test(s) used AND whether they are one- or two-sided<br>*Only common tests should be described solely by name; describe more complex techniques in the Methods section.*
☒	☐	A description of all covariates tested
☒	☐	A description of any assumptions or corrections, such as tests of normality and adjustment for multiple comparisons
☒	☐	A full description of the statistical parameters including central tendency (e.g. means) or other basic estimates (e.g. regression coefficient) AND variation (e.g. standard deviation) or associated estimates of uncertainty (e.g. confidence intervals)
☒	☐	For null hypothesis testing, the test statistic (e.g. *F*, *t*, *r*) with confidence intervals, effect sizes, degrees of freedom and *P* value noted<br>*Give P values as exact values whenever suitable.*
☐	☒	For Bayesian analysis, information on the choice of priors and Markov chain Monte Carlo settings
☒	☐	For hierarchical and complex designs, identification of the appropriate level for tests and full reporting of outcomes
☒	☐	Estimates of effect sizes (e.g. Cohen's *d*, Pearson's *r*), indicating how they were calculated

Our web collection on statistics for biologists contains articles on many of the points above.

Software and code

Policy information about availability of computer code

Data collection	DNA extractions: Qiagen QIAamp DNA Mini Kit, KingFisher™ Cell and Tissue DNA Kit. Library preparation: the protocol of Meyer & Kircher (2010), MinElute spin columns (Qiagen, Hilden, Germany), AMPure magnetic beads (Beckman Coulter™), Chromium controller instrument and reagents from 10X Genomics. Sequencing: Illumina NovaSeq (S4) and Illumina HiSeqX platforms. Cleaning and evaluation: PEAR (Zhang et al. 2014), SuperDeduper (Petersen et al. 2015), Trimmomatic v0.32 (Bolger et al. 2014), FastQC v0.11.5 (Andrews, http://bioinformatics.babraham.ac.uk/projects/fastqc/), mapDamage (Schubert et al. 2012). De novo assembly and quality control: nf-core/neutronstar analysis pipeline (Ewels et al. 2020) and BUSCOv3 (eukaryota dataset). Reads mapping and extraction of homologous sequences: BWA mem v0.7.12 (Li & Durbin 2009), BirdScanner pipeline (github.com/Naturhistoriska/birdscanner), OD-Seq (github.com/PeterJehl/OD-Seq).
Data analysis	Phylogenetic relationships: IQ-TREE (Nguyen et al. 2015), Beast2 v2.4.8 (Bouckaert et al. 2014). Estimating and analyzing genotype likelihoods: ANGSD v0.933 (NGSadmix, PCAngsd, Korneliussen et al. 2014), Cluster Markov Packager Across K (CLUMPAK, http://clumpak.tau.ac.il/index.html), R: The R Project for Statistical Computing (eigen function for PCA). Inferring divergence time: pu2fa (https://github.com/Paleogenomics/Chrom-Compare), hPSMC (Cahill et al. 2016).

For manuscripts utilizing custom algorithms or software that are central to the research but not yet described in published literature, software must be made available to editors and reviewers. We strongly encourage code deposition in a community repository (e.g. GitHub). See the Nature Portfolio guidelines for submitting code & software for further information.

Data

Policy information about availability of data

All manuscripts must include a data availability statement. This statement should provide the following information, where applicable:

- Accession codes, unique identifiers, or web links for publicly available datasets
- A description of any restrictions on data availability
- For clinical datasets or third party data, please ensure that the statement adheres to our policy

Raw sequence data are available for download from the NCBI Sequence Read Archive (SRA, BioProject PRJNA833756). Data available from the Dryad Digital Repository: xxxx

Human research participants

Policy information about studies involving human research participants and Sex and Gender in Research.

Reporting on sex and gender	N/A
Population characteristics	N/A
Recruitment	N/A
Ethics oversight	N/A

Note that full information on the approval of the study protocol must also be provided in the manuscript.

Field-specific reporting

Please select the one below that is the best fit for your research. If you are not sure, read the appropriate sections before making your selection.

☐ Life sciences ☐ Behavioural & social sciences ☒ Ecological, evolutionary & environmental sciences

For a reference copy of the document with all sections, see nature.com/documents/nr-reporting-summary-flat.pdf

Ecological, evolutionary & environmental sciences study design

All studies must disclose on these points even when the disclosure is negative.

Study description	Whole-genome sequencing of an extinct Pleistocene New World vulture, and >50 samples of recent Black Vultures.
Research sample	Nuclear genomes of an extinct Pleistocene New World vulture and >50 samples of recent Black Vultures. Fossils of the extinct vulture and the Black Vulture.
Sampling strategy	We included all Coragyps fossils found in the literature and public databases.
Data collection	Fossils and genomes.
Timing and spatial scale	Pleistocene, Holocene. New World.
Data exclusions	N/A
Reproducibility	All fossils are available in the museums where they are deposited (references are given in Table S2). Raw sequence data are available for download from the NCBI Sequence Read Archive (SRA, BioProject PRJNA833756). Phylogenomic data and genotype likelihoods are available from the Dryad Digital Repository (https://doi.org/10.5061/dryad.qz612jmjm)
Randomization	N/A
Blinding	N/A

Did the study involve field work? ☐ Yes ☒ No

Reporting for specific materials, systems and methods

We require information from authors about some types of materials, experimental systems and methods used in many studies. Here, indicate whether each material, system or method listed is relevant to your study. If you are not sure if a list item applies to your research, read the appropriate section before selecting a response.

Materials & experimental systems

n/a	Involved in the study
☒	☐ Antibodies
☒	☐ Eukaryotic cell lines
☐	☒ Palaeontology and archaeology
☐	☒ Animals and other organisms
☒	☐ Clinical data
☒	☐ Dual use research of concern

Methods

n/a	Involved in the study
☒	☐ ChIP-seq
☒	☐ Flow cytometry
☒	☐ MRI-based neuroimaging

Palaeontology and Archaeology

Specimen provenance: Peru

Specimen deposition: Swedish Museum of Natural History

Dating methods: C14

☒ Tick this box to confirm that the raw and calibrated dates are available in the paper or in Supplementary Information.

Ethics oversight: The specimens were collected in 1901-1902 and have been stored in the Swedish Museum of Natural History since then.

Note that full information on the approval of the study protocol must also be provided in the manuscript.

Animals and other research organisms

Policy information about studies involving animals; ARRIVE guidelines recommended for reporting animal research, and Sex and Gender in Research

Laboratory animals: *For laboratory animals, report species, strain and age OR state that the study did not involve laboratory animals.*

Wild animals: Coragyps atratus

Reporting on sex: The sex of the individuals was not considered in the analyses.

Field-collected samples: The samples have been collected between 1885 and 1904 and prepared as museum specimens. They are all available at the respective museum (voucher nos are given in Table S5).

Ethics oversight: The research in this paper is based on pre-existing museum collections that have been collected under appropriate permits over many decades.

Note that full information on the approval of the study protocol must also be provided in the manuscript.
